# Electrofreezing of Supercooled Water at −0.5 °C Induced by Al and Mg Electrodes via a Chemical Cooperative Process of “Ice-Making Species” and Electric Field

**DOI:** 10.1021/jacs.5c14056

**Published:** 2025-11-07

**Authors:** Shiri Dishon Ben Ami, Leah Fuhrman Javitt, Shakir Ali Siddiqui, Hagai Cohen, David Ehre, Kshatresh Dutta Dubey, Meir Lahav, Igor Lubomirsky

**Affiliations:** 1 Department of Molecular Chemistry and Materials Science, 34976 Weizmann Institute of Science, Rehovot 7610001, Israel; 2 Molecular Simulation Lab, Department of Chemistry, School of Natural Sciences, 310513 Shiv Nadar Institution of Eminence, Delhi 201314, India; 3 Chemical Research Support, 34976 Weizmann Institute of Science, Rehovot 7610001, Israel

## Abstract

Here, we report on the chemistry of electrofreezing of water near −0.5 °C as induced by aluminum and magnesium electrodes. We suggest that the processes are triggered by the action of hydrated Al^3+^ and Mg^2+^ ions or their hydrated hydroxides, as created by electrolysis and the electric field. The uniqueness of octahedral hydrated Al^3^^+^ and Mg^2^^+^ ions lies in their high polarizing power, which acidifies the coordinated water that interacts with the bulk water to create hexagonal ice-like architectures. The aluminum electrodes induce icing when used as an anode (+50 V), while magnesium electrodes induce icing either as an anode or cathode at the same voltage, where the formation of the hydroxide ions is plausible. Furthermore, molecular dynamic simulations suggest that these species induce in their surroundings, under the influence of the electric field, “ice-like” assemblies that trigger the icing process. Those simulations suggest that the order in which the species lower the entropy of the system is Al­(OH)_3_ > Mg­(OH)_2_ > Al^3+^ > Mg^2+^.

## Graphical abstract

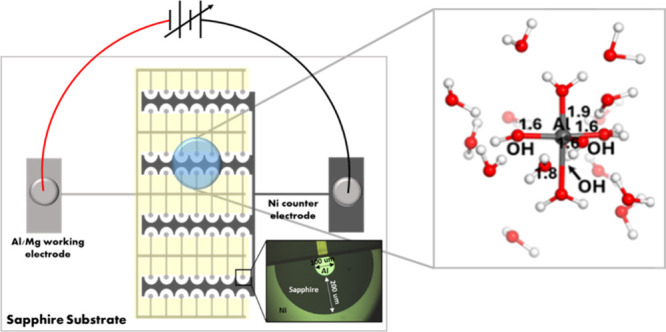

## Introduction

At standard atmospheric conditions, ice melts at 0 °C. However, water can withstand temperatures as low as −40 °C without freezing.^1^ Understanding the mechanisms that govern the freezing temperature of supercooled water (SCW) holds significance in many subfields of both pure and applied sciences, such as climate control (as rain precipitation)^2^ and food preservation.^3^

The icing temperature can be manipulated through various methods, including heterogeneous ice nucleation^4^ and the application of electric fields, a process known as electrofreezing.^5^ The first experiments in electrofreezing were performed by Dufour in 1861,^6^ and over the years, various mechanisms have been proposed to explain the phenomenon, both experimentally and theoretically.^5b,7^ Shichiri and Nagata,^8^ as well as Hozumi et al.,^9^ performed systematic investigations to clarify how different electrode materials and shapes affect the icing temperature of SCW. Their studies demonstrated that different electrodes, when subjected to electric fields, promote icing at very different temperatures. In particular, they reported that aluminum (Al) and magnesium (Mg) electrodes trigger icing close to 0 °C when serving as anodes.

To understand the mechanism of the electrofreezing extensive work was done,^10^ some experimental works suggest that nucleation of water occurs heterogeneously, near electrodes on the top of gas bubbles,^11^ via electrodewetting,^12^ or due to the influence of electric charges.^13^ Mechanistic studies suggest the raising of the icing temperature, though typically only under very high voltages and to temperatures still below −4 °C. Independently, theoretical simulations propose several mechanisms, including the alignment of water molecules by electric fields into “ice-like” architectures, resembling the polar crystal structure of the metastable polymorph ice XI, or the formation of an amorphous phase that remains stable under an electric field even above 0 °C.^14^ However, both scenarios require either extremely high electric fields (over 4 orders of magnitude larger) or lower temperatures than those applied in the experiments discussed here. For a comprehensive review see Acharya and Bahadur.^5b^ By now, the mechanism of electrofreezing using different electrodes has remained without a rational explanation. In contrast, electrofreezing experiments involving direct electric fields^15^ or inert tantalum electrodes^16^ demonstrated that electric fields alone do not significantly affect the icing process, even at lower temperatures.^15,16^ Previously, we reported that electrofreezing by cooling water droplets on surfaces of pyroelectric crystals, both in the presence and absence of the generated pyroelectric field, is a chemically cooperative process.^17^ The icing temperature can either increase or decrease, depending on the stereochemical structure of specific ions concentrated in the Stern aqueous layers, near the charged hemihedral crystal faces. In these experiments, it was found that trigonal planar ions, such as negatively charged bicarbonate or nitrite or the positively charged guanidinium, tend to concentrate near oppositely charged surfaces and interact with water molecules in a way that promotes the assembly of hexagonal, “ice-like” architectures.^17b,18^ The formation of these structures has been supported by molecular dynamics (MD) simulations.^17a^ This cooperative chemical mechanism suggests that other ions or species capable of organizing water molecules into “ice-like” assemblies that may act as kosmotropic agents in electrofreezing.^19^ A search for other kosmotropic species revealed that the octahedrally six-hydrated ions of Al^3^^+^ and Mg^2^^+^ tend, at certain concentrations, to trigger the icing of SCW within electrolytic cells at temperatures as high as −4 °C.^18b^ In contrast, other octahedral hydrated ions from the fourth row of the periodic table, such as Co^2^^+^ and Ni^2^^+^, trigger icing under neutral conditions (∼pH 7) only at or below −7 to −8 °C (Table 1).^20^ Interestingly, we found that the unique icing behavior induced by Al and Mg ions in an electric cell resembles the unusual icing properties of Al and Mg anodes, which freeze with little to no supercooling compared to anodes of other metals.^8a,9,18b^ However, the mechanism explaining how these electrodes induce icing at such high temperatures remains unclear without a mechanistic explanation.

**Table 1:** Freezing Temperatures of Water in the Absence of Voltage and under 50 V with Different Anode Materials^a^

anode’s material	freezing temperature without voltage	freezing temperature at 50 V
Al	–13 ± 3	–0.5
Mg	–20 ± 2	–0.5
Ni	–18 ± 2	<−10
Ti	–27 ± 2	<−7
Ta	–17 ± 3	<−10
Au	–27 ± 3	–8
Cr	–10 ± 2	<−10
Ag	–18 ± 3	–6

a The conditions of the experiments with the different metals as anodes were the same as for the Al and Mg anode experiments.

Building on our earlier demonstration that specific ions can act as kosmotropic species under electric fields to promote electrofreezing, we extend this mechanistic framework to explain the unusual behavior of the Al and Mg electrodes. In doing so, we bridge the ion-specific effects observed previously with electrode-induced freezing, thereby advancing our understanding of the electrofreezing mechanism.

Here, we present experimental and theoretical MD simulation studies that explain the unique mechanism of electrofreezing using Al or Mg electrodes. The unique icing properties of the electrodes of Al and Mg are brought about by Al- and Mg-hydrated ions and hydroxides generated by the electrolysis process. This process is triggered by the high polarizability that those ions bring to their coordinated and surrounding water molecules and the application of the external electric fields.

## Results

The electrofreezing experiments were performed by applying 50 V^16^ on a cell of asymmetric planar electrodes (Figure 1) deposited on a *C*-plane sapphire or quartz substrate, at −0.5 °C (electric field of ∼0.6 V/μm, using infinite cylinder approximation. At the electrode/water interface, the electric field can be much higher, reaching up to ∼500 V/μm^16^). The difference in the size of the electrodes is designed to allow a high concentration of charge at the small anode.

**Figure 1:**
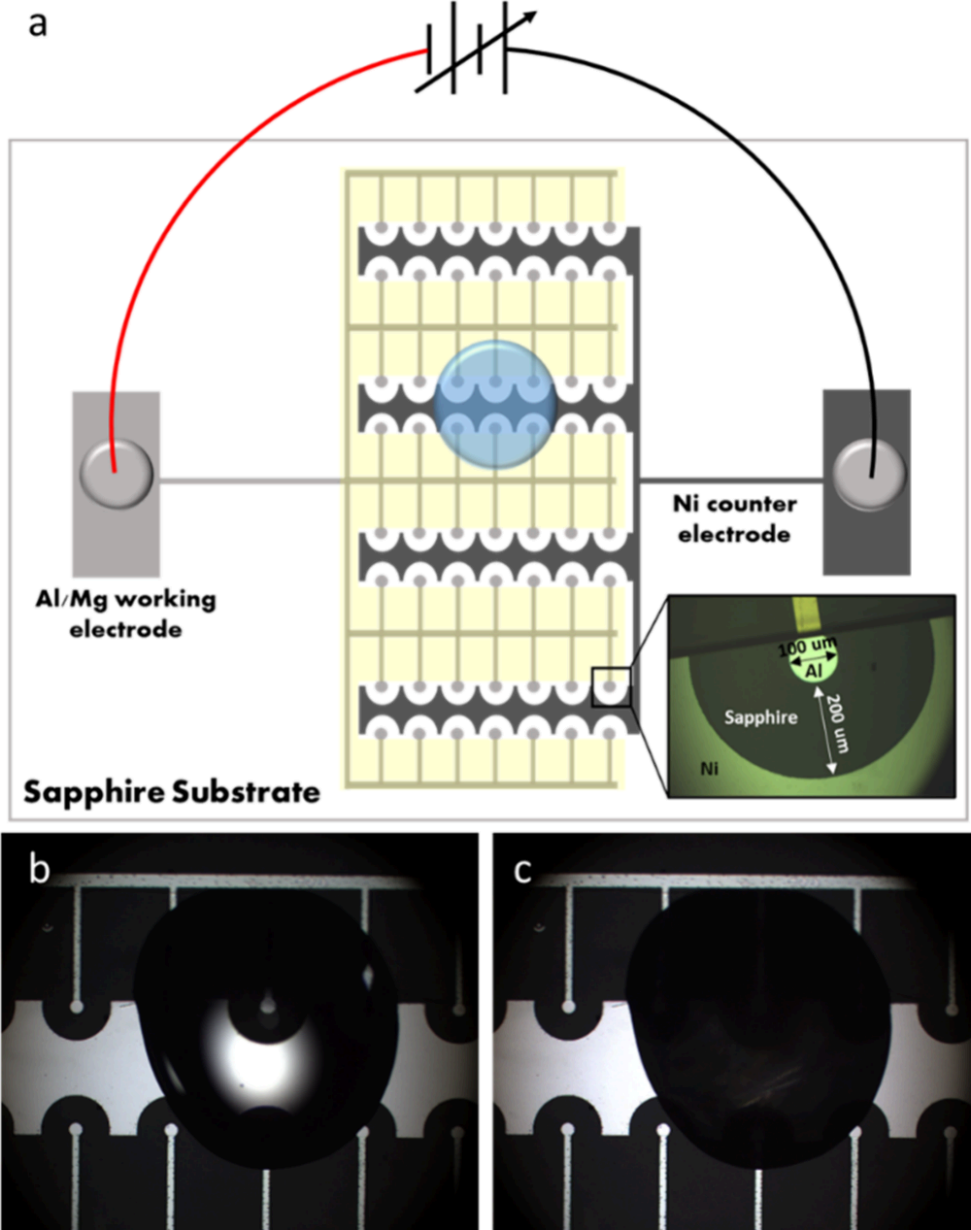
Electrodes setup used for the electrofreezing experiments. (a) Schematic representation of the electrode design: a working electrode radius of 50 μm and distance between working and counter electrode of 200 μm. An electrode array contains 56 electrode pairs, and the water droplet was placed over 4–6 electrode pairs. (b) Light microscopy photo with ×5 magnification of the experimental setup with a water droplet on the electrode before icing, the water droplet is transparent. (c) Experimental setup with water droplet on the electrode after icing, the water droplet becomes opaque.

Previous experimental studies showed that electric field alone does not induce icing without supercooling.^15,16^ Therefore, we considered a few different metals for the anode (Al, Mg, Ta, Ti, Au, Cr, Ag, and Ni). Only when the electrochemical cell consisted of Al or Mg as the anode, electrofreezing experiments showed freezing immediately upon applying the voltage without supercooling. When other metals were used as an anode, the SCW froze at much lower temperatures (below −6 °C),^18b^ as mentioned in detail in Table 1. Therefore, we chose to explore the mechanism of electrofreezing by using Al or Mg as the working electrodes. Ni was used in both cases as the counter electrode because when a high voltage is applied (up to 100 V), the SCW freezes only under −10 °C, and the Ni forms an inert stable oxide layer that remains conductive. We also considered different electrode geometries, which did not change the experimental results.

### Aluminum

To eliminate the effect of the epitaxial fit between the sample substrate and the ice, we used fused silica (quartz, amorphous) and C-plane sapphire. C-plane sapphire was chosen as a substrate since its epitaxial fit to hexagonal ice is 84%.^22^ Alternatively, quartz was chosen, because it is amorphous and has no epitaxial fit with ice. Icing experiments with no voltage applied on the sample showed that pure water freezes on Al electrodes at −13 ± 3 °C, irrespective of the substrate. In the experiments done with Al on C-plane sapphire and quartz, on both substrates, the water droplets froze immediately upon applying the voltage at −0.5 °C (in all measurements), meaning that the water freezing temperature does not depend on the substrate and the epitaxial fit between the substrate and ice.

Using an Al–Ni electrochemical cell, we found that the SCW freezes at −0.5 °C only under positive bias, namely, only when the Al acts as an anode, not as a cathode. Similar experiments were also performed with pure D_2_O. The freezing temperature of D_2_O is 3.8 °C;^23^ therefore, as expected, the D_2_O droplet froze at 3 °C when 50 V was applied.

The process occurring at the Al anode is Al → Al^3+^ + 3e, which implies that the Al^3+^ ions have an important role in promoting the ice nucleation process. This is supported by our previous work showing that the hydrated Al^3+^ ions can induce icing in the presence of an electric field.^18b^ It is also possible that hydroxide ions generated at the cathode migrate toward the anode, where they react with Al^3+^ ions to form hydrated Al­(OH)_3_. Those species may also promote ice nucleation before eventually precipitating from the solution.

The threshold positive voltage needed for freezing the SCW is ∼30 V for Al, which is relatively high. This result agrees with our hypothesis that Al ions must be introduced into near-surface water. To do so, a sufficiently high voltage must be applied to break the continuous and stable native oxide layer that covers the electrodes and to oxidize the metal without causing mechanical shock to the system.

To learn more about the species created during the application of voltage and responsible for the promotion of the freezing event, we applied 50 V at 1 °C (above the freezing temperature of water) for a few seconds, turned off the voltage, and cooled the water slowly (at a rate of 2 °C/min). The water did not freeze at −0.5 °C and instead froze below −10 °C. This result shows that the species promoting the nucleation appears when the voltage is turned on and disappears immediately when the voltage is turned off or that the species only induces ice nucleation in the presence of an electric field. Nevertheless, it shows that any modification of the electrode’s surface that happens due to the application of voltage does not promote the nucleation of the ice in the absence of the electric field.

### Surface Analysis

To find evidence that the breaking of the Al electrode is necessary to induce electrofreezing, scanning electron microscopy (SEM) images of the electrode were taken before applying the voltage on the sample and after applying 50 V and freezing of the droplet (then, the droplet was heated to 1 °C to melt the ice, and the water was evaporated in the nitrogen environment) (Figure 2). These images clearly show the breakdown of the anode material (Figure 2c before and Figure 2e after), and energy-dispersive X-ray analysis (EDAX, Bruker XFlash/60) shows that Al disappeared from the anode after applying the voltage. Figure 2d shows the Al before applying voltage (red), in which the working electrode appears to be whole, while Figure 2f (after applying voltage) shows that the Al from the electrode disappears (red). Dissolution of the Al electrode results in differences between Figure 2d (before applying voltage), showing no oxygen from the substrate around the electrode (blue), and Figure 2d (after applying voltage), where oxygen from the substrate appears (blue). This is because, when the Al electrode is undamaged, it blocks the oxygen signal from the quartz substrate from reaching the EDAX detector. The Ni (Figure 2d,f, green) remains unchanged before and after the application of voltage.

**Figure 2:**
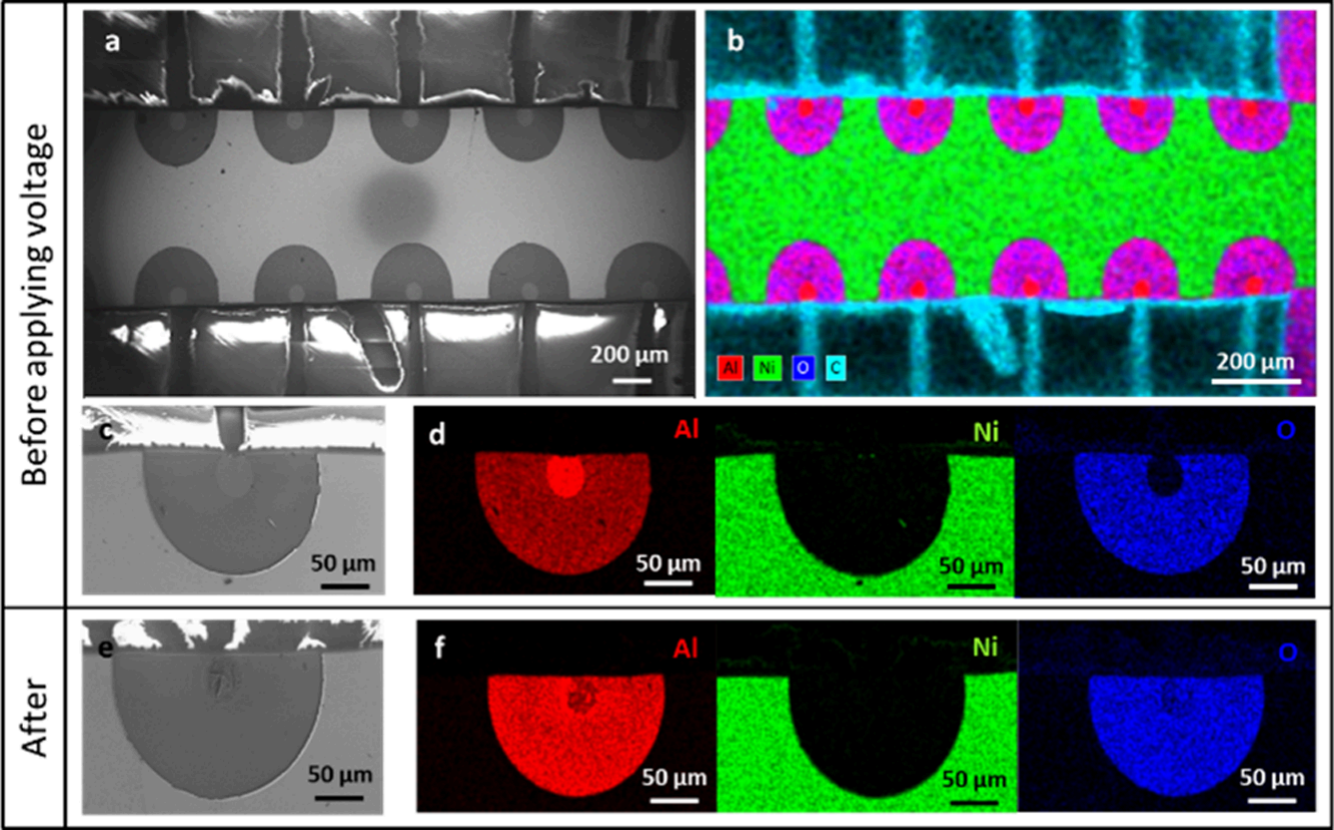
(a) SEM and (b) EDAX image of the Al (red) working electrode and Ni (green) counter electrode on sapphire substrate (Al_2_O_3_) before applying voltage. (c) Higher magnification of one electrode pair before applying voltage. (d) Elemental EDAX maps of the same electrode pair, showing Al (red), Ni (green), and oxygen (blue), before voltage application. (e) SEM image of the electrode pair after voltage application. (f) Elemental EDAX maps of the electrode pair after voltage application, showing Al (red), Ni (green), and oxygen (blue). These images clearly demonstrate the breakdown of the Al anode upon applying voltage.

Figure 3 shows several representative X-ray photoelectron spectroscopy (XPS) energy-filtered maps of the Al–Ni system deposited on a quartz substrate. All maps are shown here on a log scale to conveniently present weak signals superimposed on a large background. Figure 3a presents a reference area not exposed at all to water. The two electrodes are seen very clearly, and no diffusion is observed. The Ni-electrode also appears bright in the Al map because of an overlapping Ni 3p signal.

**Figure 3:**
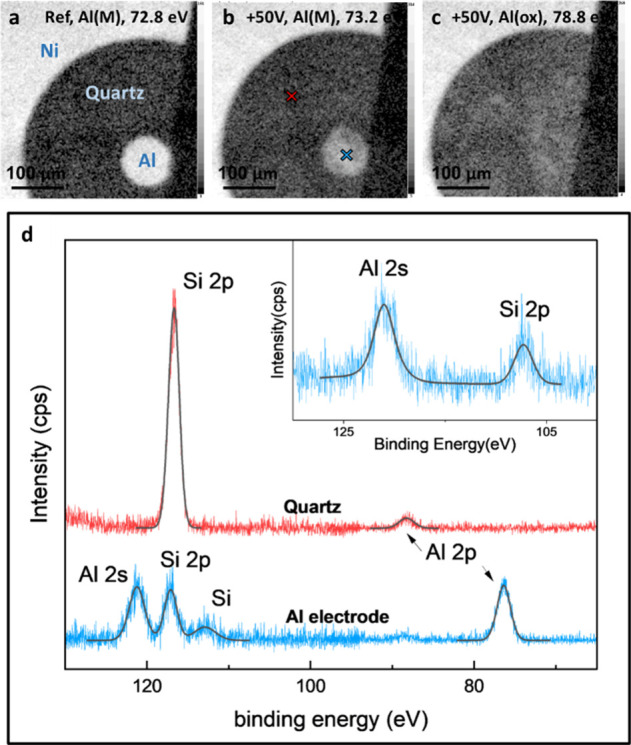
XPS elemental maps and small-spot spectra of the Al–Ni electrode system. (a) An Al map of the reference configuration (not exposed to water). (b) Al map at the forward bias configuration, encountering only negligible charging. (c) Al map at the forward bias configuration, encountering rather large charging shifts. (d) Spectra recorded with an aperture of 55 μm from (blue) the center of the Al electrode and (red) a central point on the quartz area between electrodes. The inset in panel (d) shows a corresponding spectrum with an aperture of 15 μm at the Al electrode center, verifying no residual signals from the electrode periphery. Note in the inset spectrum, the appearance of the Si signal was attributed to a hole in the electrode.

To capture the presence of Al ions near the electrode after applying 50 V and the diffusion toward the negatively charged Ni electrode by XPS mapping, we had to consider the beam-induced charging effects encountered at the insulating quartz surface. In Figure 3b,c, we show two Al 2p maps, taken at different binding energy windows (73.2 and 78.8 eV, respectively), thus differentiating between grounded (on electrode) and charging-shifted (on quartz) signals. At the interelectrode space, i.e., the quartz surface, clear nonmetallic Al signals are seen (see Figure 3b,c), shifted to higher binding energies due to (1) location-dependent positive charging and (2) the standard oxidation-related chemical shift. Note that the surface of the central electrode is highly oxidized (or hydroxide-coated, probably >12 nm thick, hence, no metal signal could be resolved there). Interestingly, clear evidence for the formation of holes in this electrode is found, due to the out-diffusion of Al (see dark spots in Figure 3b). This latter point is verified by both nonuniformity in the Al maps and, complementarily, by the appearance of a Si signal from the quartz underneath the electrode (see inset spectrum in Figure 3d).

Figure 3d shows spectra of the system after applying 50 V, recorded (1) on the Al central electrode and (2) at the interelectrode space, as indicated in Figure 3b (in blue and in red). The energy window used here covers several lines: the Ni 3p (not appearing at the points shown here), Al 2p, Si 2p, and Al 2s core levels. On both electrodes, negligible charging artifacts are found, whereas the quartz surface gives rise to remarkable location-dependent charging, up to 14 eV in magnitude. Note that the location-dependent charging encountered here significantly complicates the spectrum. However, in turn, it introduces location-dependent information that is exploited in our analysis.^24^

To better visualize where the nucleation process begins, electrofreezing experiments using solutions of 1:2 sucrose:water were performed, because sucrose is known to impede the growth of ice crystals.^25^ The experiments were performed at −10 ° C, to be below the icing temperature of the sucrose solutions. By adding sucrose to the solution, we determined that the ice nucleation always begins at or near the surface of the Al anode (Figure 4). After the nucleation begins, each nucleus grows until they connect, and the whole droplet freezes.

**Figure 4:**
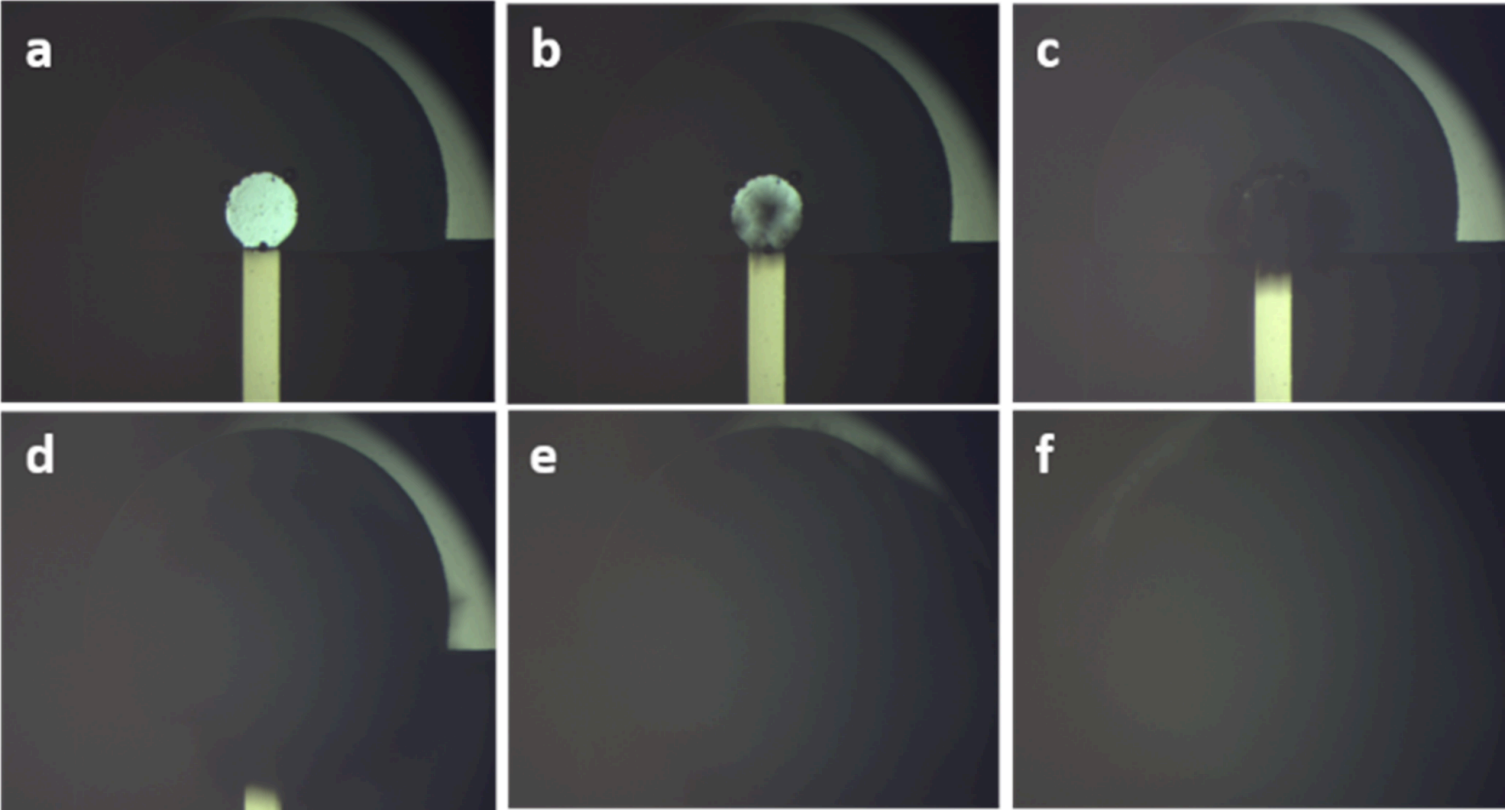
Icing of 1:2 sucrose solution on Al–Ni electrode pair under light microscope with ×20 magnification. Ice nucleation begins at or near the Al anode and grows until the entire droplet undergoes a phase change to ice.

### Molecular Dynamics Simulations

To provide mechanistic insight into the experimental observations, we extended our study with molecular dynamics (MD) simulations. We performed different sets of the MD simulations with Al^3+^ and Al­(OH)_3_ in the aqueous medium and a separate simulation for ice nucleation without any metal ions. Each simulation was performed using an explicit model of alumina electrodes, as shown in Figures S2 and S3.

Previous theoretical works^18b^ show that the ice nucleation process is observed at the external electric field (EEF) up to 1 V/Å, which is much larger than the experimental conditions used here. Therefore, we first optimized the EEF for the electrofreezing process by using different values of EEF and performing MD simulations to achieve an optimized scale. In our previous work, we found that an EEF of about 0.2 V/Å, coupled with the TIP4P/ICE water model, provides a significant insight into nucleation, and therefore, all data reported below are at an EEF of 0.2 V/Å and at −3.15 ± 3 °C using the same water model.

The MD simulations for each system without an external electric field show no nucleation (Supporting Information, Figures S4 and S5). Additionally, in pure water, MD simulation did not show hexagonal packing until 100 ns of the simulations, even in the presence of an external electric field of 0.2 V/Å. This result aligned with the previous finding that metal ions are necessary for ice nucleation.^18b^ Interestingly, the MD simulations with the metal ion Al^3+^ and an EEF of 0.2 V/Å clearly show the hexagonal packing of the systems, as shown in Figure 5a and Figure S6. However, the pattern of nucleation (in terms of simulation time) was significantly different for different systems. The simulation indicated that in the aqueous solution with Al^3+^, nucleation commences after ∼7–8 ns and onward.

**Figure 5:**
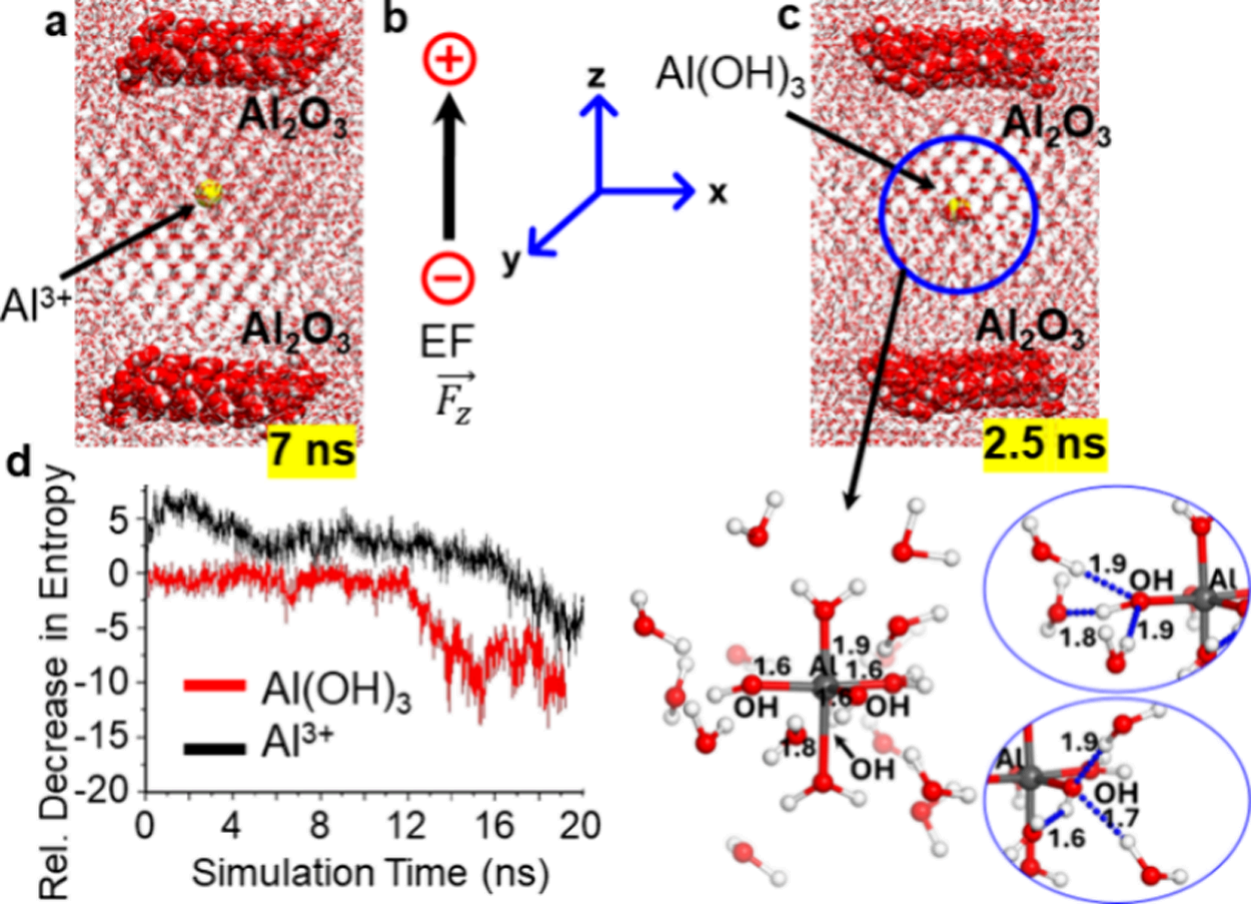
Molecular dynamics (MD) simulation snapshots showing hexagonal packing behavior under an external electric field of 0.2 V/Å: (a) Al^3^^+^ and (c) Al­(OH)_3_ near the Al_2_O_3_ surface. (b) Cartesian axes indicating the electric field (*F_z_*) applied perpendicularly to the surface, following the Gaussian convention. (d) Relative decrease in the configurational entropy of water molecules within 5 Å of metal ions, calculated from radial distribution functions. Water exhibits a larger entropy decrease near Al­(OH)_3_ than Al^3^^+^, indicating a greater structural ordering (Al­(OH)_3_ > Al^3^^+^). Zoomed-in views highlight hexagonal coordination of water molecules around aluminum species.

Based on this observation, we performed a separate MD simulation with Al­(OH)_3_ under similar EEF conditions. For Al­(OH)_3_, surprisingly, the nucleation is faster and starts the formation of the ice-like hexagons as early as ∼2.5–3 ns of the simulation time (see Figure 5c and Figure S7). These results revealed a notable acceleration in the onset of nucleation, suggesting that the presence of three OH^–^ moieties indeed contributed to the enhanced nucleation rate. Upon closer examination of the MD trajectory in the presence of Al­(OH)_3_ vis-à-vis Al^3+^ in water, it is clearly apparent that the OH^–^ moiety plays a pivotal role in expediting the growth of ice-like hexagonal packings. Each OH^–^ ion of Al­(OH)_3_ can facilitate the growth of three ice-like hexagons. The rdf shows a peak at ∼2.7–2.8 Å (see Supporting Information, Figure S11), closely matching an O–O distance of 2.75–2.80 Å in crystalline ice Ih, corresponding to an H-bond length of ∼1.78 Å.^26^ This provides important mechanistic insight that the OH^–^ ion could be crucial in ice nucleation processes by formatting the multiple hexagonal structures, resulting in the faster growth of hexagons. To ensure the robustness of our findings, we corroborated these results using ab initio molecular dynamics (AIMD) simulations, which confirmed the pivotal role of OH^–^ groups in accelerating the nucleation process under similar electric field conditions (see Supporting Information, Text S1 and Figure S10).

Moreover, when considering the combined effect of OH^–^ and H_2_O molecules within a [Al­(OH)_3_(H_2_O)_3_] cluster, it becomes evident that these two groups (water and OH^–^) can collectively polarize more surrounding water molecules. This polarization effect, in turn, enhances the propensity for hexagonal packing to grow in numerous directions simultaneously, thereby speeding up the process of ice nucleation when compared to alternative scenarios. In essence, the concerted action of OH^–^ and H_2_O molecules within Al­(OH)_3_ serves as a catalyst for rapid and efficient ice nucleation, unveiling insights into the underlying mechanisms governing this phenomenon. The coordination of OH^–^ groups significantly impacts both entropy reduction and nucleation enhancement, with the nucleation-promoting effect following the trend: Al­(OH)_3_ > [Al­(OH)_2_]^+^ > [Al­(OH)]^2^^+^ > Al^3^^+^, underscoring the critical role of OH^–^ coordination in the ice nucleation process (see Supporting Information, Text S2).

To further substantiate these findings, we analyzed the entropy of water molecules surrounding the Al metal ions, as shown in Figure 5d. This analysis was based on the radial distribution function of water molecules within a 5Å radius of the metal ions (see Supporting Information, Text S3). From the graph, we can see that the decrease in the entropy around Al­(OH)_3_ is sharper as compared to that of Al^3+^. These results clearly show a reduction in entropy, confirming the significant impact of the metal ions on the packing of water molecules, implying a more organized configuration of water molecules in the vicinity of the metal ions, which likely contributes to the pronounced ice nucleation.

### Magnesium

Icing experiments without external voltage showed that pure water freezes on the sample with Mg electrodes at −20 °C ± 2 °C, irrespective of the substrate. In the Mg–Ni electrochemical cell, the SCW freezes under positive or negative bias at −0.5 °C (in all measurements). The process occurring at the Mg anode is: Mg → Mg^2+^ + 2e and as was already mentioned, Mg^2+^ has been shown to act as an “ice-making” ion in the presence of an electric field. It is also possible that hydroxide ions generated at the cathode, migrate to the anode, and react with the hydrated Mg^2+^ to form hydrated Mg­(OH)_2_, which may induce ice nucleation before precipitating out of the solution. SEM images of the Mg anode show breaking of the electrode (Figure 6a–c). The threshold voltage for icing to occur at the Mg anode is 45 V ± 5. Notably, ice nucleation occurs also when Mg is the cathode. This is in contrast to previously published results by Shichiri and Nagata,^8a^ which showed that Mg, as the cathode, does not induce icing when the counter electrode is Au. In this case, much higher voltages were used than the voltages used in our experiments. We have found that Au electrodes cannot withstand over 20 V, so we speculate that in the previously reported experiments, the electrode system may have broken down before ice nucleation occurred. In our experiments, icing occurs at the Mg electrode upon the application of a −40 V ± 5 V pulse.

**Figure 6:**
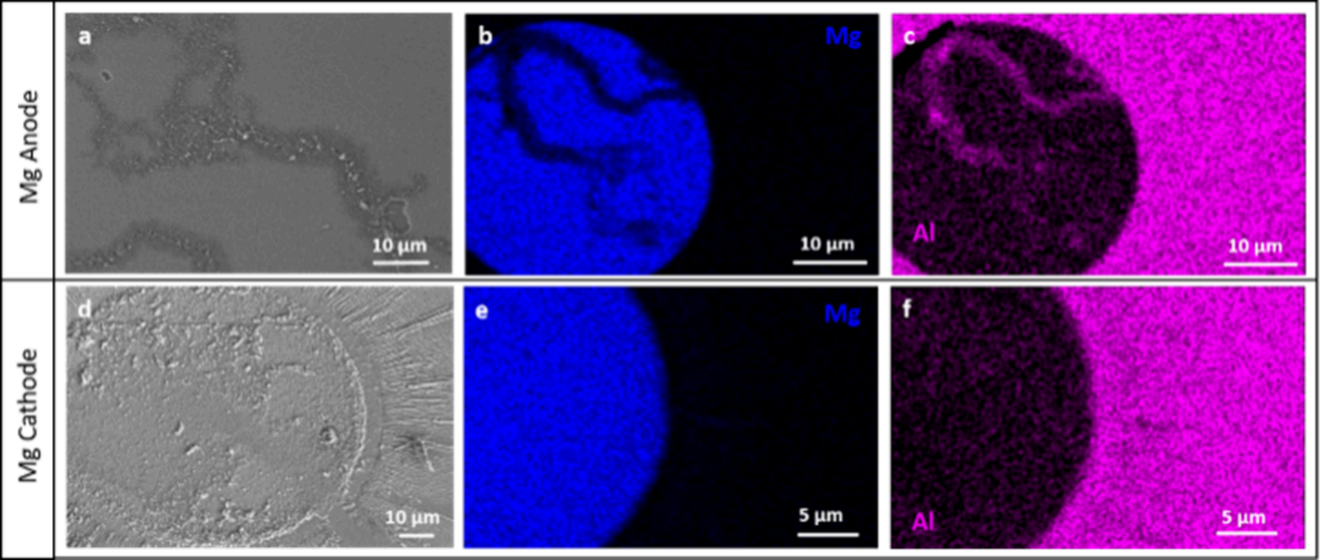
(a–c) SEM and EDS images of the Mg (blue) anode after the application of voltage and (d–f) SEM and EDS images of the Mg cathode after the application of voltage. The electrodes were deposited on a sapphire substrate, and the Al (magenta) visible in the EDS images is from this surface.

### Surface Analysis

The Mg electrode after negative bias displays a unique halo of material surrounding it, and the original electrode appears to be porous (Figure 6). While EDAX was unable to identify the surface restricted chemical composition of the halo (Figure 6e,f), XPS mapping shows that it contains Mg (Figure 7). SEM images, see Figure 6d–f, show that the Mg cathode reacts thoroughly before inducing ice nucleation.

At the cathode, MgH_2_ can be formed upon the application of voltage through the formation of H^–^ ions, which can react with the Mg.^27^ MgH_2_ readily reacts with water to form hydrated Mg­(OH)_2_, which induces ice nucleation before precipitating out of the solution. Another possible mechanism for ice nucleation at the cathode is that in the presence of water, Mg metal reacts to form a stable Mg­(OH)_2_ layer. Since this layer no longer consists of hydrated ions, it is not likely to induce ice nucleation. Upon the application of a large negative voltage, the layer mechanically breaks, and new metallic Mg is introduced to the water, which can react to form hydrated Mg­(OH)_2_, which can then induce ice nucleation. In both of these mechanisms, hydrated Mg­(OH)_2_ is responsible for the ice nucleation.

Figure 7 shows representative energy-filtered XPS maps (on a linear intensity scale) of the Mg–Ni system. The original size and shape of the electrodes are identical to the ones shown in Figure 3a, recorded from an “as received” system. The maps in Figure 7b,c were recorded at the forward bias configuration, showing the Mg 2p and O 1s energy windows, where the latter depicts the specific peak associated with bonding to Mg. The corresponding spectra are given in Figure 7a: a blue curve taken at the center of the Mg electrode and a red curve taken at the “center” of the interelectrode sapphire space. Corresponding spots are indicated in Figure 7b. As already explained for the Al–Ni system, charging at the interelectrode domains is significant and, therefore, the Al 2p sapphire peak is shifted to a higher binding energy, by about 3 eV (see red curve in Figure 7a). The Mg diffusion is not necessarily very apparent in Figure 7b, but its existence and gradual decay in concentration toward the Ni electrode are significant. More reliably, small-spot spectra (55 μm aperture) recorded across the sapphire region show undoubtedly the related Mg signals (Figure 7a, red curve). Complementarily, the (much stronger) O 1s peak allows improved mapping of the Mg diffusion, and indeed, Figure 7c shows an “enlarged electrode” due to the high amounts of near-electrode Mg–O species. Unfortunately, due to the location-dependent charging (due to the insulating nature of the substrate), differentiation between Mg oxide and Mg hydroxide was challenging, based on these spectra.

**Figure 7:**
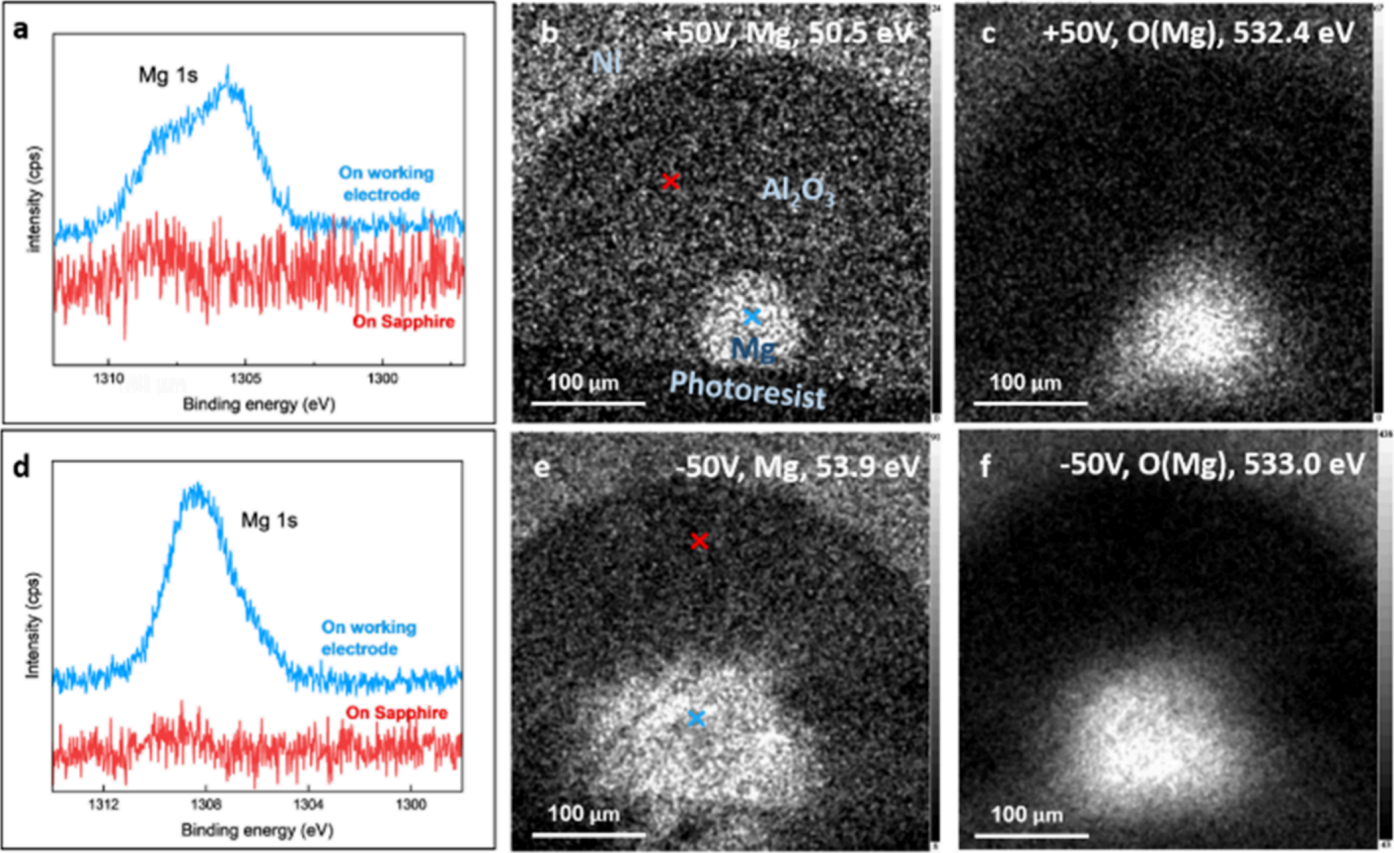
XPS small-spot spectra and elemental maps (filter energy indicated in each) of the Ni–Mg system. (a) Mg 1s spectra for the forward bias configuration, recorded with an aperture of 55 μm from (black) the center of the Mg-electrode and (red) a central point on the sapphire spacer. (b, c) Elemental maps in the forward bias, the (b) Mg 2p, and the Mg-bonded component of the (c) O 1s. (d) Representative small-spot spectra of the reverse bias configuration (e, f) Elemental maps in the reverse bias configuration, (e) Mg 2p and the (f) Mg-bonded O 1s component. Note the significant enlargement of the “electrode-related” central Mg spot, due to diffusion.

Figure 7d–f corresponds to the reverse bias configuration. Mg diffusion into sapphire domains is significantly greater in this case, as is easily seen in Figure 7e (the Mg map) and in Figure 7f (the map of Mg-bonded O 1s component). Note that the sapphire charging shifted the Al signal (Figure 7d red curve; expected to appear around 74.5 eV), which helps us to better identify the energy of Mg residues in those domains. Here too, we could not reliably differentiate between Mg-oxide and Mg-hydroxide species.

### Molecular Dynamics Simulation Results

Similar to the MD simulations performed with Al^3+^ ions and Al­(OH)_3_, we extend our simulations including Mg^2+^ and Mg­(OH)_2_ in the solvent. As discussed above, it is likely that Mg­(OH)_2_ is present at (or near) the surface of the Mg electrode. Our results show that in the aqueous solution of Mg^2+^, the nucleation lagged and commenced during ∼13–14 ns (Figure 8a and Figure S8). This result matches with the previous finding for the ions in the bulk solution.^18b^ Furthermore, in the presence of Mg­(OH)_2_ under the same EEF, we observed an even faster hexagonal rearrangement of the molecules surrounding Mg­(OH)_2_. In this case, nucleation starts around 3–4 ns after simulations (Figure 8b and Figure S9). In fact, by comparing the nucleation patterns of Mg^2+^ vis-à-vis Mg­(OH)_2_, we see that the nucleation in Mg­(OH)_2_ is quicker by 10 ns. This early onset of nucleation in the presence of Mg­(OH)_2_ may be attributed to the presence of two hydroxide ions, which may enhance the interaction with water molecules, facilitating quicker ice nucleation compared to Mg^2+^. This shows behavior similar to what we had observed in the case of Al­(OH)_3_. Here, the two OH^–^ and four H_2_O molecules interact mutually and facilitate ice-like hexagons in a faster manner than that of the hydrated Mg^2+^ ion. Furthermore, the hexagonal packing observed in the hydrated Mg­(OH)_2_ (Figure 8b and Figure S9) is also more clear and significant vis-à-vis the other two.

**Figure 8:**
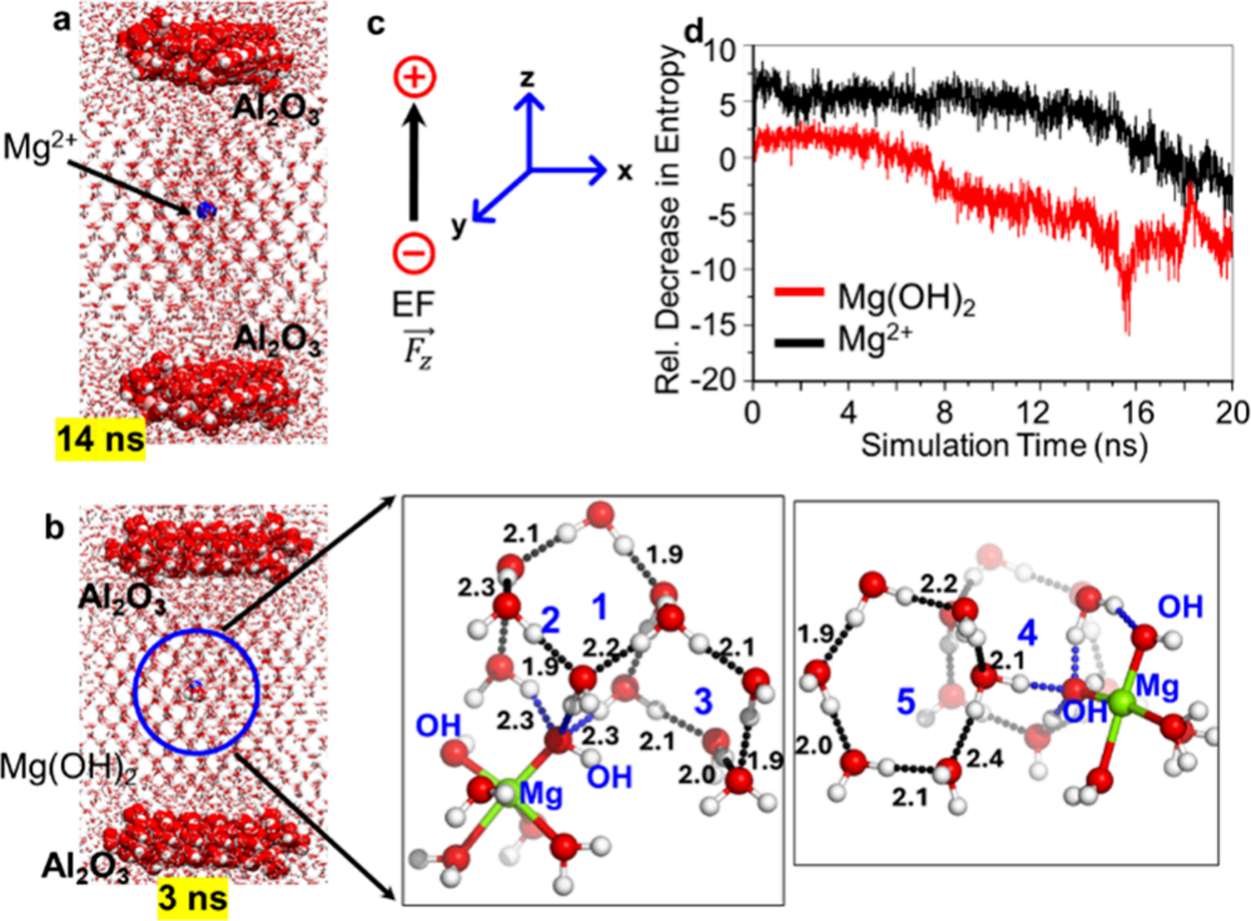
Depiction of the hexagonal packing behavior observed in all scenarios under an external electric field of 0.2 V/Å during molecular dynamics (MD) simulations with (a) Mg^2+^ and (b) Mg­(OH)_2_. (c) Cartesian axes indicating the electric field (*F*_*z*_) applied perpendicularly to the surface, following the Gaussian convention. (d) Relative decrease in entropy of water molecules surrounding metal ions, calculated based on the radial distribution function within a 5 Å radius. The hierarchy of entropy decrement observed is Mg­(OH)_2_ > Mg^2+^.

To further substantiate these findings, we analyzed the entropy^28^ of water molecules surrounding the Mg^2+^ and Mg­(OH)_2_ as shown in Figure 8d. These results were again in the complementarity that the entropy decrease near the Mg­(OH)_2_ is more than that of the Mg^2+^. These results clearly show the reduction in entropy, confirming the significant impact of the metal ions on the packing of water molecules, implying a more organized configuration of water molecules in the vicinity of the metal ions, which likely contributes to the pronounced ice nucleation. Icing when Mg­(OH)_2_ is present in solution shows a further reduction in entropy when compared to icing experiments in the presence of the Mg hydrate or the absence of either of these species (Figure 8d, Figure S12). This implies a more organized configuration of the water molecules, similar to the case of Al.

### Entropy and Electrostatic Stabilization Energy Calculations

From the entropy calculation of the simulated systems, we found the following order to Al­(OH)_3_ > Mg­(OH)_2_ > Al^3+^ > Mg^2+^ (see Supporting Information, Figures S12 and S13). This hierarchy underscores the varying degrees of influence exerted by metal hydroxides and metal ions on the surrounding water molecules, further elucidating the importance of an ion’s ability to order the surrounding water molecules on ice nucleation dynamics.

To further investigate the effects of the external electric field on water molecules surrounding Al^3+^, Mg^2+^, Mg­(OH)_2_, and Al­(OH)_3_, we compared the interaction of the dipole moment with external electric field and the electrostatic stabilization energy, Δ*E*, of these water molecules. This analysis involved calculating the stabilization of the dipole moment of water molecules (μ_*z*_) located within a 5 Å radius of the metal ions. The electrostatic stabilization energy (ΔE) was determined as the difference caused in dipole moment with and without the application of the EEF. It gives the stabilization of water dipoles near the metal (hydroxides) due to the EEF. The results of this calculation are summarized in Table 2. Notably, near the metal ions Al^3+^ and Mg^2+^, the dipole moment increased substantially from −3.35 and −3.02 D to 34.38 and 24.67 D when subjected to the EEF. Similarly, for water molecules near Mg­(OH)_2_ and Al­(OH)_3_, the dipole moment increased from −23.04 and −31.12 D, respectively, to 73.69 and 89.54 D, respectively, when subjected to the electric field, showing that the water molecules present near the metal hydroxides are more aligned and stabilized. Since the electrostatic stabilization energy (Δ*E* in kcal/mol) is directly correlated with μ_*z*_ (Δ*E* = 4.8·μ·*F*_*z*_·cos θ), we believe that the stabilization effect will increase linearly with the dipole moment for a constant EEF. These findings underscore the critical role of the external electric field in interaction with the water dipoles, reorienting them and influencing the stabilization of water molecules, thereby affecting the overall dynamics of ice nucleation.

**Table 2:** Calculation of the Electrostatic Stabilization Energy (Δ*E*) for Water Molecules within a 5 Å Radius of Metal Ions and Their Hydroxides, Both without and with an Applied Electric Field of 0.2 V/Å

water molecules in 5 Å radius	μ_*z*_, dipole moment, without EEF [Debye]	external electric field (EEF), *F*_*z*_ [V/Å]	μ_*z*_ with an EEF of –0.2 V/Å [Debye]	Δ*E* [kcal/mol]
near Al^3+^	–3.35	–0.2	34.38	–33.00
near Al(OH)_3_	–31.12	–0.2	89.54	–85.96
near Mg^2+^	–3.02	–0.2	24.67	–23.68
near Mg(OH)_2_	–23.04	–0.2	73.69	–70.74

## Discussion

The topic addressed here is part of a broader study aimed at understanding why the icing temperature of SCW under an electric field, when induced by electrodes of different metals, diverges dramatically, even when the experiments are performed under similar electric conditions (Table 1).

To induce ice nucleation of SCW at a given temperature, it is necessary to stabilize supermolecular architectures of a given size that resemble the structure of ice.^13e,17,29^ Such domains are rarely found within pure bulk water, where molecules interact primarily by hydrogen bonds. Thus, to induce icing at a given temperature, the ice has to be created with the assistance of different auxiliary agents. The critical size of ice nuclei is estimated experimentally and theoretically as 25 Å^2^.^30^

More specifically, through experiments and MD simulations, we present a mechanistic model suggesting a chemical cooperative process driven by an external electric field and the operation of the Al and Mg hydrated and hydrated hydroxide clusters, which operate as kosmotropic units. These molecular clusters form “ice-like” architectures that serve as nucleating centers for ice crystallization.

Among metal ions, Al^3^^+^ and Mg^2^^+^ stand out in their ability to act as kosmotropic ions due to the following unique properties: (1) From an entropic perspective, Al^3^^+^ and Mg^2^^+^ order coordinated water molecules into stable octahedral hydration structures.^31^ (2) Although different ions yield octahedral hydrated ions, the metal ions of Al^3^^+^ and Mg^2^^+^ stand out due to their valency and small ionic radius that induce strong polarizing power, which converts them to acidic Lewis acids.^32^ Al^3^^+^ lowers the local pH to ∼2–3 and Mg^2^^+^ to ∼5–6.^18b^ The acidic coordinated water molecules of Al and Mg can interact with the surrounding bulk water molecules, which according to the Bro̷nsted–Lo̷wry principle, are considered as basic, thus create supramolecular water architectures distinct from these found in pure bulk water.^33^ Ions of the fourth layer of the periodic table, such as Co^2+^ and Ni^2+^, do not display such polarizability, and their coordinated water molecules are not acidic.^20b^ Consequently, they cannot serve as kosmotropic ions for the electrofreezing of SCW. (3) During electrolysis, hydrated hydroxide species of Al are formed near the anode, and hydrated hydroxide species of Mg formed both near the anode and near the cathode.

Based on these considerations, we propose the following mechanism: during the application of an electric field, the Al or Mg oxide layer, covering the anode, mechanically breakdown. This causes the Al^3+^ and Mg^2+^ ions to be released to the water, as can be supported by the SEM and EDAX images. As a result, there is a high concentration of Al^3+^ or Mg^2+^ in the solution, especially near the electrode, as can be seen in the XPS results. In addition, the electric field leads to the splitting of the water at the cathode, which yields hydroxide ions. These hydroxides can be attracted to the positively charged anode and react with Al^3+^ or Mg^2+^ to generate hydroxide species.

MD simulations suggest the formation of the ice-like clusters near both electrodes, induced either by the hydrated ion or by the hydrated hydroxide species. These clusters then promote icing. In addition, these simulations suggest that the initiation of the icing near the electrode in the presence of hydrated Al^3+^ is faster and starts after 7 ns, whereas in the presence of the hydrated Mg^2+^, it starts after 14 ns. Moreover, the simulations suggest that the hydrated hydroxides induce icing even more efficiently than the hydrated ions (2.5 ns for Al­(OH)_3_ and 3 ns for Mg­(OH)_2_). The ice nucleation can be initiated at the electrode surface itself, as demonstrated with the experiments of the sucrose. However, those experiments do not exclude the option that the icing might also be initiated at the aqueous layer near the electrode and promoted by the electrodes’ surface and water interactions.

Since Mg can react with water at low temperatures at both electrodes, even without an electric field to form Mg­[(OH)_2_(H_2_O)_4_], Mg electrodes trigger icing when operating both as an anode and as a cathode. In contrast, metallic Al only reacts with water above 40 °C^34^ and, therefore, induces icing only when functioning as an anode.^35^

Taken together, these results reveal that the interplay between electrode composition, ion hydration properties, and electric field creates a cooperative mechanism for ice nucleation with Al and Mg uniquely suited to facilitate this process through their kosmotropic behavior.

## Conclusions

Here, we show experimental work and MD simulations on electrofreezing near the melting point of ice induced by Al and Mg electrodes. We suggest a cooperative chemical process between an external electric field and the hydrated Al^3+^ or Mg^2+^ ions or their hydroxylated species (which are created during the electrolysis process).

Those ions have two unique properties: first, they arrange coordinated water molecules in an octahedral configuration, which provides an entropic advantage. Second, they engender strong polarizability, which acidifies those water molecules and converts them into Lewis acids. During the electrochemical process, they interact with OH^–^ ions created near the cathode as a result of the electrolysis of water and conversion into a Lewis base. Those Lewis species are stronger acids or bases in comparison to the acidity or basicity of the surrounding bulk hydrated layers.^37a^ Consequently, when those coordinated acidic or basic water, or OH^–^ units, interact with the surrounding water molecules with the assistance of the electric field, they create new supramolecular water architectures, different from those present in bulk water and created by the hydrogen bond of the water molecules.

MD simulations suggest that during such interactions, supramolecular hexagonal architectures are created, which consequently serve as nuclei for the icing process almost without supercooling. Moreover, the simulations also suggest that the hydrated hydroxides clusters induce icing even more efficiently than the hydrated ions (nucleation is faster in 4.5 ns for Al­(OH)_3_ and 11 ns for Mg­(OH)_2_).

Under the same experimental conditions, electrodes of the fourth layer of the periodic table, such as Co and Ni, where the coordinated water around the created ions are not acidic, do not promote acid–base interaction that affects the icing temperature as Al or Mg electrodes.^33^

The cooperative effect of species formed under an applied electric field and hydrating water molecules is a mechanism of electrofreezing. However, other mechanisms remain to be discovered, such as the formation of an epitaxial fit under an applied electric field.^20a^

Finally, the present mechanism displays likeness to the Hofmeister^36^ and the water fluidity^37^ effects in which ions induce changes in the bulk water that influence macroscopic properties of water.

## Methods

### Electrode Preparation

All electrodes were deposited on c-plane sapphire or quartz substrates.

Al electrode was fabricated by e-beam deposition (Telemark) of 200 nm of metallic Al (99.999%), followed by photolithography (photoresist S1805, MA/BA6 Karl-Suss mask aligner) and etch (Al etchant, Transene Company Inc.).

The Mg electrode was fabricated by 200 nm Mg deposition using magnetron sputtering (ATC Orion Series Sputtering System, AJA International Inc.), followed by photolithography (photoresist S1503) and etch (25:75:1 H_2_O:ethylene glycol:HNO_3_).

Ni electrode was prepared by photolithography (photoresist AZ2514 or AZ4562) followed by e-beam deposition of Ni (99.999%) and lift-off using acetone.

The protective layer made from photoresist AZ4562 was obtained by photolithography. Wires were attached to the electrodes using silver conductive paint (SPI).

### Ice Nucleation Measurements

To determine the SCW freezing point, a 50 μL droplet of boiled, ultrapure distilled water (Millipore, 18.2 MΩ·cm) was placed on the sample. The sample was then cooled on a Peltier stage from room temperature down to the specific temperature of the measurement at a rate of 2 °C/min, using an INSTEC mK2000 temperature controller. After the freezing event, the sample was heated slowly (0.5 °C/min) to room temperature in order to monitor the melting point. A K-type thermocouple connected to a Keithley 2110 5 1/2 Digit Multimeter was used to measure and record the temperature of the sample during the experiments. Correction of melting point to 0 °C, and the freezing point, accordingly, is used to eliminate artificial shifts in measured temperature originating from the thermocouple. The freezing temperature was monitored by a light microscope (Zeiss AXIO Imager.M2.m) connected to a complementary metal-oxide-semiconductor (CMOS) BlueFOX3 camera.

Each icing on Al electrodes and Mg electrodes with and without voltage were repeated at least 50 times in each condition, each experiment was done on new and clean electrodes pairs. The errors in the freezing temperature and threshold voltage are the standard deviations.

The voltage applied on the samples using a power source, either Alpha Dielectric Analyzer (Alpha Novocontrol) or Keysight B2912A Precision Source/Measure Unit.

### X-ray Photoelectron Spectroscopy (XPS)

XPS measurements were performed on a Kratos AXIS-Ultra DLD spectrometer, using the monochromatic Al kα anode at source power levels of 15–75 W. Energy-filtered images (in the Kratos parallel imaging mode) and small-spot spectra were acquired in order to explore the spatial and spectral characteristics of the interelectrode space, as well as the very surface of each electrode. Electrodes were usually fabricated on sapphire substrates; however, for the Al electrode experiments, quartz substrates were used as well. For imaging, a parallel detection mode was used with “field of view 2” and pass energy of 160 eV, thus enabling lateral resolution of about 5 μm. Complementarily, small-spot spectra were acquired at selected locations using aperture sizes of 15–55 μm. Beam-induced effects were investigated by comparing early scans with repeated ones, including longer (up to 36 h) exposures on a given spot.

In most cases, both the anode and cathode arrays were grounded, such that signals originating from any of the electrode sites could be spectrally resolved from those signals originating from the sapphire (or quartz) surface. The reason for this capability stems from the fact that charging artifacts were markedly larger at the insulator domains, namely, in the interelectrode surface areas. As demonstrated previously^24,38^ and realized in the present system too, superior lateral resolution can frequently be achieved by exploiting the differential charging artifacts on heterogeneous surfaces. Best results in this context were obtained under positive-charging conditions, with which spectral differentiation of a few eV in magnitude was established between “on-electrode” and “on-insulator” spots. Finally, by varying the charging conditions (i.e., increased X-ray flux and/or activation of the electron flood gun, eFG), consistency of the interpretation was cross-validated. It should be stressed that the improved sensitivity to ion diffusion effects was, to some extent, subject to the accuracy in the evaluation of chemical oxidation states, such as in the differentiation between hydroxide and oxide states of the reacted metal.

### Computational Details and Methods

We used molecular dynamics (MD) simulations (both classical and AIMD) in the presence and absence of an external electric field and electrostatic stabilization energy due to the electric field in order to study the effect of the electric field on ice nucleation. Below are the detailed descriptions of each method used in the study:

#### System Setup

Initially, we took the crystal structure of α-alumina (α-Al2O3) from the Crystallography Open Database (COD)^39^ with a CIF code of 9007634.cif as extensively used in the previous studies.^22,40^ The unit cell parameters and the 3D arrangement of the alumina molecules as a sheet are shown in Figure S2. These are obtained from experimental observations.^41^ Here, the α-Al_2_O_3_ slabs are placed opposite each other in the simulation box while dipped in bulk water (Figure S3). They are placed opposite each other to avoid the unphysical electric fields that can arise due to their thickness.^42^ The outer O atoms are capped with H to avoid any false long-range electric fields.^40^ In all the system preparation and the simulation, the distance between the center of mass of the two electrodes has been taken as 50Å, and the metal ions and the metal hydroxides are placed exactly between the two electrodes, which is 25Å from each. We have used CLAYFF force field^43^ to model the interactions between the α-Al_2_O_3_ electrodes and the bulk water molecules. We have used TIP4P/ICE water model,^44^ which has the normal melting point 270 ± 3K.^39^ In our simulations containing charged like Al^3+^ species, we added an appropriate number of Cl^–^ counterions to maintain overall charge neutrality.

#### Molecular Dynamics (MD) Simulation Setup

Following the parametrization of the system, we initiated a minimization process aimed at rectifying any unfavorable contacts and refining the geometry. This involved a sequential application of 5000 steps utilizing the steepest descent method, followed by an additional 5000 steps employing the conjugate gradient approach. During this minimization, the Al atoms of the α-alumina electrodes were restrained to their position. Temperature adjustment was done through a gradual annealing process over 50 ps within the NVT ensemble, with constraints imposed on the electrodes and the metal ions and metal hydroxides. Subsequently, a 1 ns density equilibration simulation was conducted under the NPT ensemble, maintaining a constant temperature of 270 K and pressure of 1.0 atm. Temperature and pressure were regulated using the Langevin thermostat^45^ with a collision frequency of 2 ps and the Berendsen barostat^46^ with a pressure relaxation time of 1 ps. Throughout the density equilibration phase, weak restraints were applied under periodic boundary conditions until a uniform density was achieved. Upon reaching uniform density, all of the previously applied restraints from the heating and density equilibration stages were removed. Furthermore, to maintain the proper position of the electrodes, we retained a weak restraint over the Al atoms of the electrodes and metal ions. The systems underwent a further equilibration period of 3 ns before commencing production MD simulations. 100 ns production MD simulations were performed for each system. To ensure simulation reliability, three replicas were executed for each production run, each initialized with distinct initial velocities. The external electric field was applied uniformly and exactly perpendicular to the plane of the two electrodes. Hydrogen atoms were constrained using the SHAKE algorithm,^46b^ while long-range electrostatic interactions were managed using the PME method.^47^ All simulations in absence and presence of external electric fields were performed utilizing the GPU version of the AMBER22 package.^48^ Following the production runs, a trajectory analysis was performed using the CPPTRAJ module of the AMBER package. Visualization of trajectories was facilitated using VMD software,^49^ and figures illustrating our findings were prepared using VMD and PYMOL.^49,50^

#### Electrostatic Stabilization Energy

Interaction and stabilization energies were then computed using a specific formula, incorporating Δ*E* as the stabilization energy in kcal·mol^–1^, representing LEF (*F*_*z*_) intensity in V·Å^–1^, indicating dipole moment magnitude (μ) in Debye, and θ denoting the angle between LEF and dipole moment vectors, with calculations adhering to GAUSSIAN convention:^51^

$$\Delta E=4.8(\vec{F_{z}}\cdot \vec{\mu }\to )=4.8|\vec{F_{z}}|\cdot |\vec{\mu }|\cdot \cos\;\theta$$

#### Ab Initio Molecular Dynamics (AIMD) Simulations

AIMD simulations were performed to capture the polarization effects and subtle electronic responses of water molecules under strong electric fields, which are inaccessible to classical nonpolarizable force fields. All AIMD calculations utilized the B3LYP functional combined with a 6-31G basis set for all of the atoms, chosen for its balance between computational efficiency and accuracy in capturing hydrogen-bonded interactions and dipole reorientation dynamics. We took 48 water molecules and an ion hydroxide in our simulation box. Simulations were conducted on GPU in a cubic simulation cell containing water molecules and the relevant ionic species using QUICK QM package^52^ in AMBER.^53^ A time step of 1 fs was used to ensure accurate integration of the electronic structure and atomic forces.

To simulate an external electric field (EEF), given that QUICK lacks a dedicated EEF keyword, a pair of charged circular plates was created using TITAN-code.^54^ Each plate consisted of 25 concentric rings of dummy atoms with a 2 Å spacing between rings. These plates were positioned 7 Å from an aluminum (Al) ion centered in the simulation box (see Supporting Information, Figure S10). The charges on the dummy atoms were set to generate a uniform electric field of 0.2 V/Å within the solvated system. Electric field was applied parallel to the *z*-axis to examine field-dependent changes in water orientation, hydrogen bond network dynamics, and ionic hydration shell behavior. The electronic structure was updated at each time step to capture polarization, allowing for a detailed examination of dipole moments and proton transfer events within hydration shells. To stabilize the computational demand, we employed temperature scaling using Langevin dynamics with the collision frequency 2 ps^–1^ that set to maintain the system near ambient temperature (∼273 K), mimicking experimental conditions. The total simulation time for AIMD trajectory was 100 ps, as shorter time scales are typical in AIMD but sufficient to observe initial electrofreezing effects and proton dynamics. Analyses included radial distribution functions and dipole moment distributions under field and no-field conditions.

## Supplementary Material



## Notes

Summary of results of freezing temperatures of water in the absence of voltage and under 50 V; XPS Ni map (at binding energy = 862 eV) of the electrode system at reverse bias configuration; schematic representation of MD simulation setups for the different systems (pure water, Al^3+^ in water, Mg^2+^ in water, and Mg­(OH)_2_); MD simulation in the absence of electric field; close look at the evolution of ice-like hexagonal formation near the Al^3+^ metal ion, Al­(OH)_3_, Mg^2+^, and Mg­(OH)_2_; crystallographic structure of α-Al_2_O_3_; AIMD simulations of Al­(OH)_3_ in external electric field (EEF); impact of partially hydroxylated aluminum species on entropy and nucleation; relative entropy calculation from MD (PDF)

## Abbreviations

SCW: supercooled water
Ta: tantalum
Ni: nickel
Al: aluminum
Mg: magnesium
SEM: scanning electron microscopy
EDAX: energy-dispersive X-ray spectroscopy
XPS: X-ray photoelectron spectroscopy
MD: molecular dynamics
EEF: external electric field
AIMD: ab initio molecular dynamics
