# Supplementary material for: Electrofreezing of Supercooled Water at −0.5 °C Induced by Al and Mg Electrodes via a Chemical Cooperative Process of “Ice-Making Species” and Electric Field
Source: J Am Chem Soc. 2025 Nov 7;147(49):45119–31. doi: 10.1021/jacs.5c14056 (PMC12703723; doi:10.1021/jacs.5c14056)
Supplement: Supplementary file 1 [file ja5c14056_si_001.pdf]

Supporting Information

Electrofreezing of Super Cooled Water at -0.5°C Induced by Al and Mg electrodes via a Chemical Cooperative process of 'Ice Making Species' and Electric Field

Shiri Dishon Ben Ami[1*], Leah Furman Javitt[1*], Shakir Ali Siddiqui[2*], Hagai Cohen[3], David Ehre[1], Kshatresh Dutta Dubey[2], Meir Lahav[1] and Igor Lubomirsky[1]

[1]Department of Molecular Chemistry and Materials Science, Weizmann Institute of Science, Rehovot, Israel

[2]Molecular Simulation Lab, Department of Chemistry, School of Natural Sciences, Shiv Nadar Institution of Eminence, Delhi, India

[3]Chemical Research Support, Weizmann Institute of Science, Rehovot, Israel

*These authors contributed equally to this work

Correspondence authors: KDD (kshatresh.dubey@snu.edu.in), ML (meir.lahav@weizmann.ac.il), IL (igor.lubomirsky@weizmann.ac.il

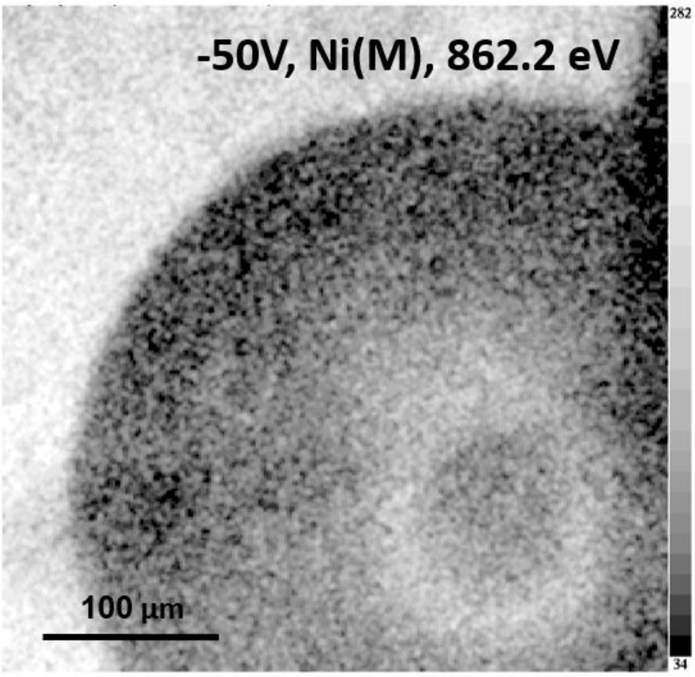

Figure S1 - XPS Ni map (at binding energy = 862 eV) of the electrodes system at reverse bias configuration (-50V on the smaller working electrode). In this case, as expected electrochemically, since the Ni experienced a positive bias, significant Ni diffusion towards the central electrode is observed. Note that this diffusion is very efficient across the quartz interelectrode space, such that Ni-accumulation around the Al electrode takes place.

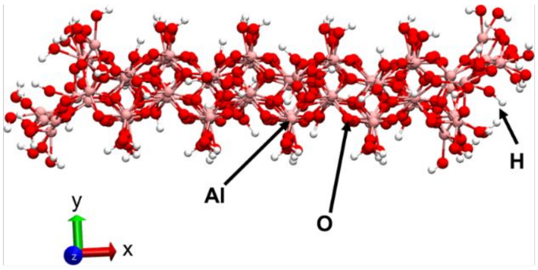

- Taken from **Crystallography Open Database (COD)**
- Coordinates ID: **9007634.cif**
- a 4.754 Å
- b 4.754 Å
- c 12.99 Å
- α 90°
- β 90°
- γ 120°

Figure S2 - The crystallographic structure of α-Al_2O_3 sheet/slab and the necessary crystallographic information taken from the experimental observations.

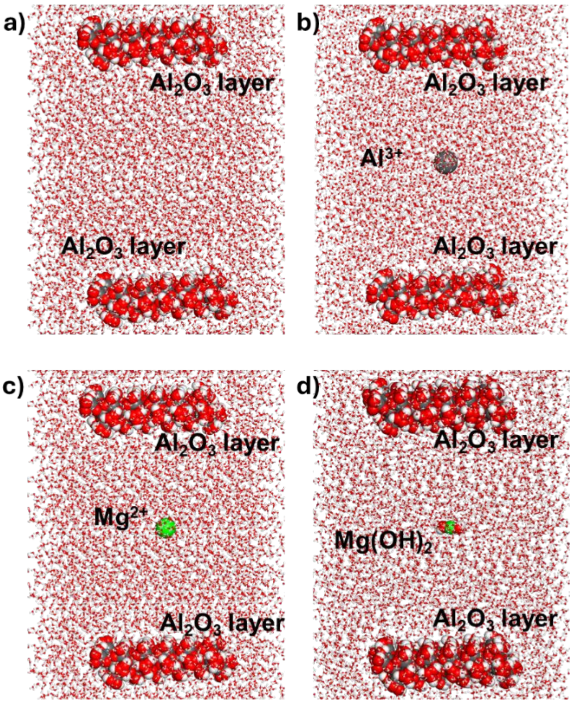

Figure S3 - Schematic representation of MD simulation setups for the different systems: (**a**) water, (**b**) Al^{3+} in water, (**c**) Mg^{2+} in water, and (**d**) $Mg(OH)_2$ in water, positioned between the alumina electrodes. The color codes for atoms are as: O atoms are represented in red, H in white, Al in gray, and Mg in green.

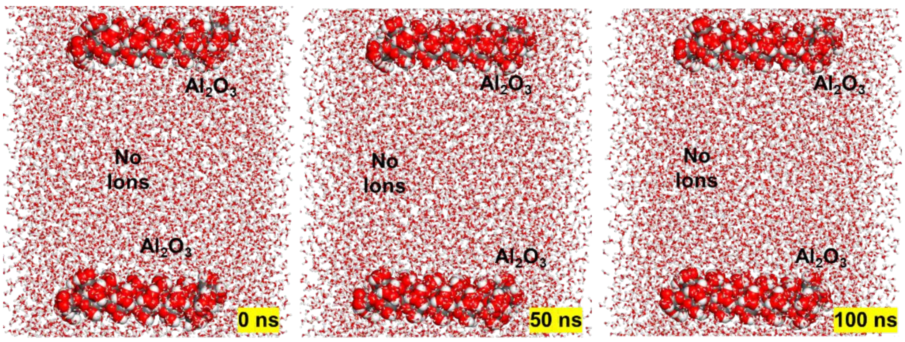

Figure S4 - No nucleation was observed during the MD simulations in the absence of external electric fields. This is the scenario when no ions were present between the two alumina electrodes.

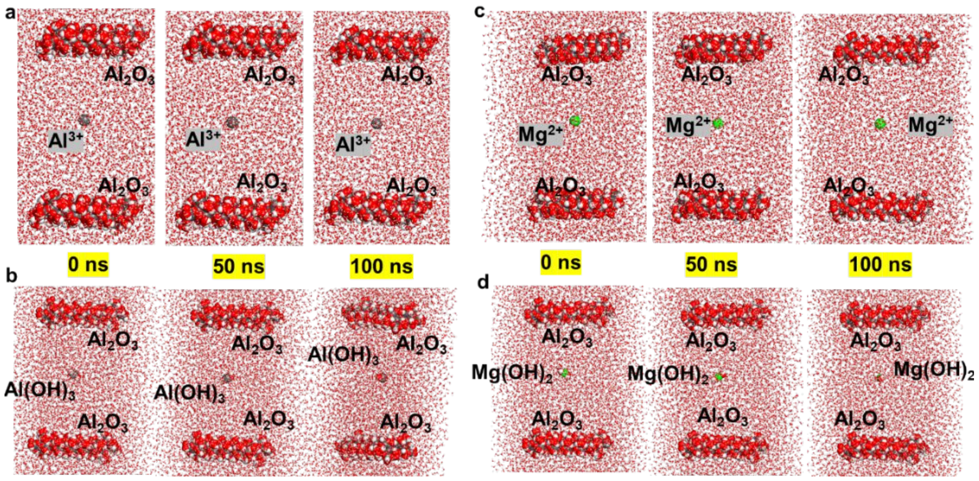

Figure S5 - No nucleation was observed during the MD simulations in the absence of external electric fields. These are the scenario when (**a**) Al^{3+}, (**b**) $Al(OH)_3$, (**c**) Mg^{2+} and (**d**) $Mg(OH)_2$ were present between the two alumina electrodes.

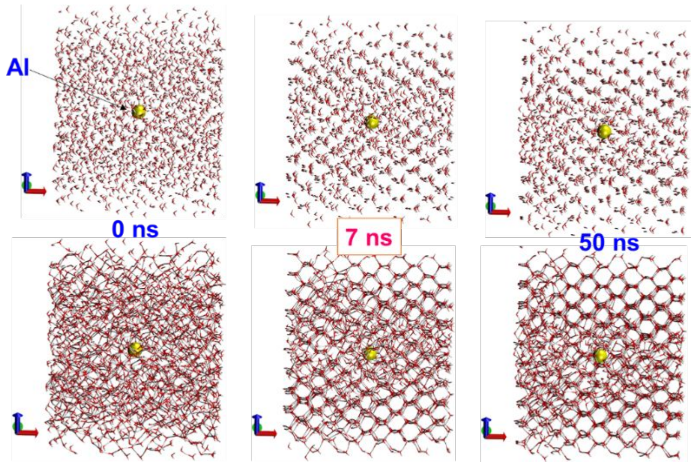

Figure S6 - A close look at the evolution of ice-like hexagonal formation near to the Al^{3+} metal ion. Electrodes are omitted for clarity.

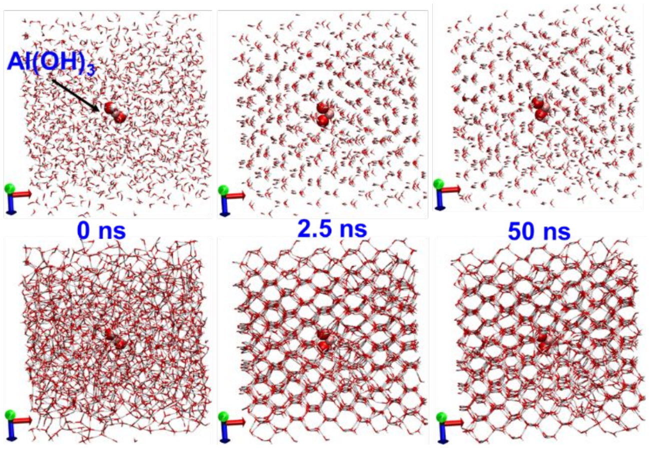

Figure S7 - A close look at the evolution of ice-like hexagonal formation near to the $Al(OH)_3$. Electrodes are omitted for clarity.

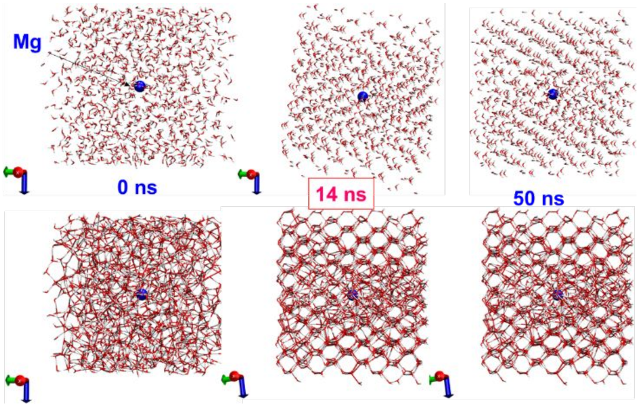

Figure S8 - A close look at the evolution of ice-like hexagonal formation near to the Mg^{2+} metal ion. Electrodes are omitted for clarity.

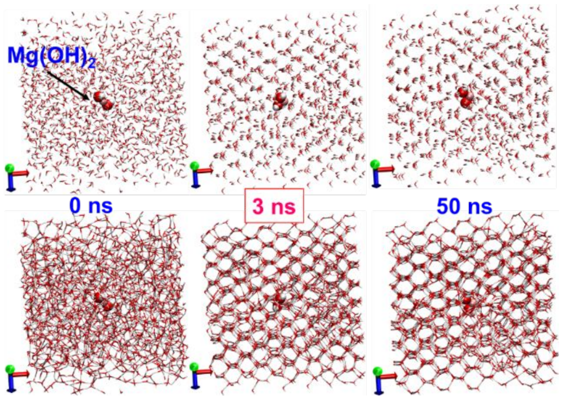

Figure S9 - A close look at the evolution of ice-like hexagonal formation near to the $Mg(OH)_2$. Electrodes are omitted for clarity.

Text S1 - ab initio (AIMD) Simulations of $Al(OH)_3$ in External Electric Field (EEF):

We have performed ab initio molecular dynamics (AIMD) simulations using the B3LYP/6-31G level of theory. In these simulations, we modeled a system of 48 water molecules with one $Al(OH)_3$ in the presence of a 0.2 V/Å external electric field (see **figure S10**).

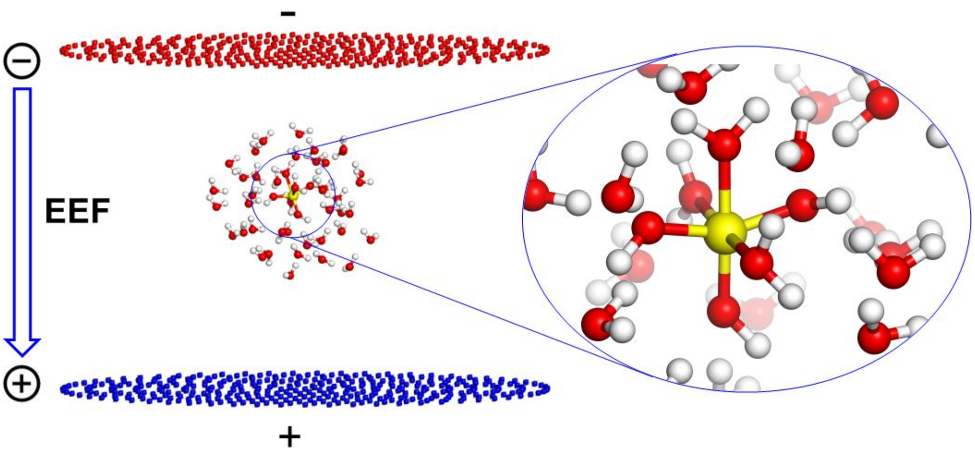

Figure S10 - Simulation setup for applying an external electric field in the AIMD with QUICK in AMBER. For electric field vector, the GAUSSIAN convention is used. A pair of charged circular plates, each consisting of 25 concentric rings of dummy atoms with 2 Å spacing, was generated using TITAN-code. The plates were positioned 7 Å from an aluminum (Al) ion at the center of the simulation box. Blue concentric circles represent positive charges, while red circles indicate negative charges. The charges on the dummy atoms were set to produce a uniform electric field of 0.2 V/Å.

Our AIMD results show that while the OH^- group of $Al(OH)_3$ does interact with nearby water molecules, accepting a proton to form a new OH^- group, this process does not lead to any breakdown of the water structure or any significant change in the electrofreezing behavior. Rather, the system maintains charge balance, and the water network remains stable. Notably, the chain-like reorganization of Al-ligated water molecules to generate OH^- groups is transient and does not appear to disrupt the overall electrofreezing process. These findings suggest that while proton hopping may occur locally within the hydration shell of the ions, it does not significantly alter the larger-scale dynamics of electrofreezing as observed in our experiments.

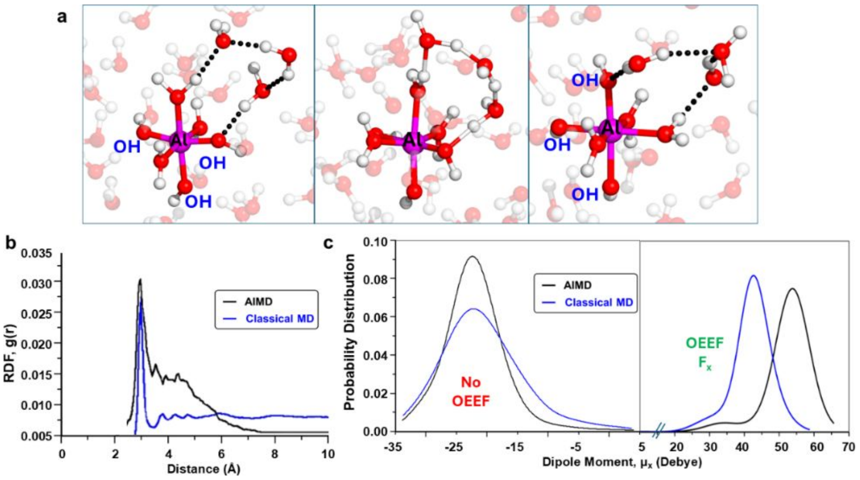

Figure S11 - (**a**) Structural snapshots of the $Al(OH)_3$ complex with water molecules from AIMD simulations (hydrogen bonding in dashed black lines). (**b**) Radial distribution function g(r) for Al–O distances in water, comparing AIMD (black) and classical MD (blue). (**c**) Probability distribution of dipole moment μ_x. Left: Without an external electric field (OEEF), Right: Under an applied electric field (F_x). μ_x is defined as the x-component of the total dipole moment of the water + $Al(OH)_3$ system.

Moreover, we have additionally compared the classical simulations and AIMD. In the radial distribution function (RDF) plot, AIMD shows a sharper peak at around 2 Å, indicating a more structured solvation shell compared to classical MD, where the RDF peak is broader and slightly shifted. This difference suggests that AIMD captures quantum effects in the solvation environment around the $Al(OH)_3$ complex, providing a more accurate representation of hydrogen bonding interactions. In the dipole moment distribution without an external electric field, AIMD predicts a lower and narrower distribution, which reflects electronic polarization effects not accounted for in classical MD as expected. Under an applied electric field, both AIMD and classical MD simulations show a shift in the dipole moment distribution to higher values, but the AIMD response is more pronounced as the increased dipole moments have more probability.

Though this comparison emphasizes the superiority of the AIMD, including the polarization effects, the overall conclusion qualitatively remains the same in classical simulations as well.

Text S2 - Impact of Partially Hydroxylated Aluminum Species on Entropy and Nucleation:

To explore the role of intermediate species like $[Al(OH)_2]^+$ and $[Al(OH)]^{2+}$ in influencing entropy and nucleation behavior, we extended our calculations to include these species using the same methodology (classical MD) applied to $Al(OH)_3$, $Mg(OH)_2$, Al^{3+}, and Mg^{2+}. The entropy of water molecules surrounding these species was assessed based on their radial distribution functions (RDF) within a 5 Å radius. The results (**figure S11b**) reveal a consistent trend of decreasing entropy with fewer OH^- groups coordinated to the aluminum ion, following the order: $Al(OH)_3 > [Al(OH)_2]^+ > [Al(OH)]^{2+} > Al^{3+}$. The RDF plots (**figure S11a**) show the spatial distribution of water oxygen atoms around aluminum hydroxides, with the first peak intensity near ~2 Å diminishing as the number of OH^- groups decrease. This reduced coordination tendency for higher-charged species indicates a decrease in hydration shell structuring. A similar trend was observed for magnesium species, where $Mg(OH)_2 > Mg^{2+}$ in entropy reduction and nucleation efficiency.

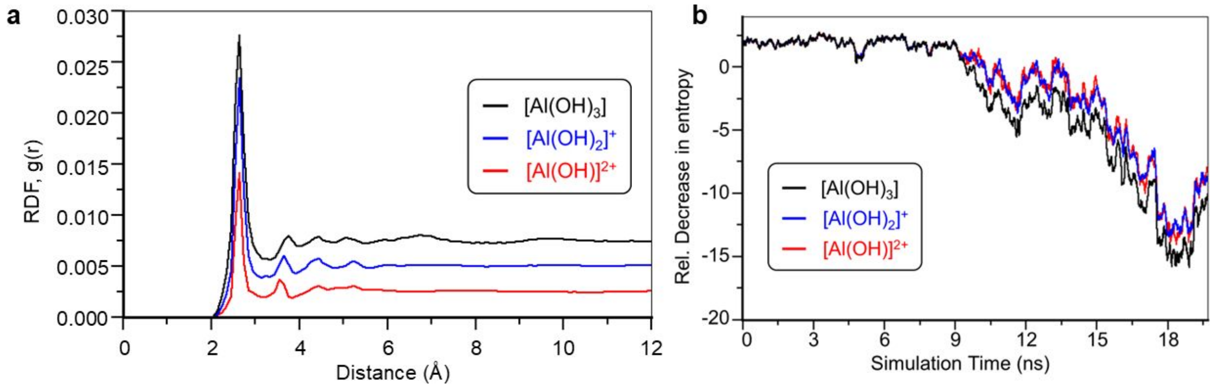

Figure S12 - (**a**) Radial distribution function for different Al hydroxides. (**b**) Comparing the relative decrease in entropy of water molecules surrounding metal ion, calculated based on the radial distribution function (RDF) within a 5 Å radius, on varying the number of OH groups from $Al(OH)_3$ to specifically $[Al(OH)_2]^+$, $[Al(OH)]^{2+}$.

In terms of nucleation behavior, partially hydroxylated species demonstrated proportional nucleation enhancement relative to their OH^- coordination. While the effect is less pronounced than $Al(OH)_3$, it remains significantly greater than that of bare Al^{3+}. This supports the hypothesis that OH^- groups play a vital role in nucleation by promoting entropy reduction and enabling the formation of nucleation-favorable local structures. These findings corroborate the trends discussed in the main text, reinforcing the critical role of OH^- coordination in ice nucleation processes. This data provides deeper insight into the mechanisms by which hydroxylated species influence the dynamics of nucleation under external electric fields.

Text S3 - Relative local structuring analysis from MD:

The analysis of local water structuring from the radial distribution function (RDF) of water molecules was performed using an in-house Python script based on the MDAnalysis library. We computed the RDF of water oxygen atoms (Ow) around the metal hydroxide (Mg-HYD/Al-HYD) groups over the simulation trajectories.

The RDF, $g(r)$, describes the probability of finding a water molecule at a distance r from the metal center. In an NPT ensemble, $g(r)$ can be related to the potential of mean force (PMF) via the relation:

$$w(r) = -RT\ln g(r)$$

Where, $w(r)$ is the PMF, R is the gas constant, and T is the temperature.

In our method, RDF Calculation: For each frame, the distances between water molecules and metal hydroxide were calculated and binned to generate g(r). Trend Analysis: The evolution of $g(r)$ over time was used to track relative changes in local water structuring during the simulation. Importantly, the analysis was aimed at observing relative structuring trends rather than calculating absolute thermodynamic entropy. Therefore, direct entropy values were not extracted. Instead, we interpret changes in RDF profiles qualitatively, noting that sharper and more defined RDF peaks indicate increased local order around the metal center. To compare different systems, we monitored the time evolution of RDF features and calculated relative changes from initial frames as a reference.

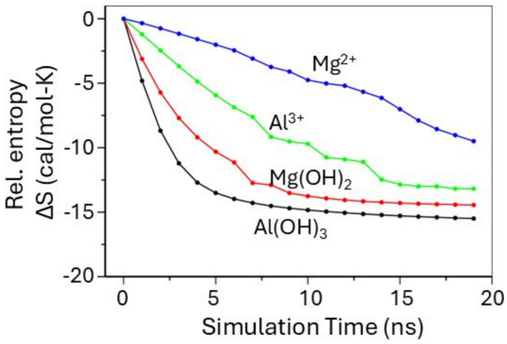

Figure S13 - Relative entropy, ΔS (cal/mol-K) calculated for $Al(OH)_3$, $Mg(OH)_2$, Al^{3+}, Mg^{2+} calculated using quasi-harmonic entropy calculations. The order in which the species lowers the entropy of the system is $Al(OH)_3$>$Mg(OH)_2$>Al^{3+}>Mg^{2+}

Additionally, quasi-harmonic entropy calculations were carried out using the cpptraj module of AmberTools to estimate internal entropic contributions from atomic fluctuations in the MD trajectories. Prior to analysis, trajectories were RMS-fitted to a reference frame to eliminate global translational and rotational motion, and solvent molecules (away than 5 Å of metal ion) and ions/elctrodes were removed using standard cpptraj preprocessing commands. A mass-weighted covariance matrix of atomic positional fluctuations was then computed using the matrix covar command. This matrix was diagonalized to obtain eigenvalues corresponding to principal modes of motion. Frequencies were derived from these eigenvalues assuming harmonic behavior, and the corresponding entropy

contributions were computed using the quantum harmonic oscillator approximation. The total quasi-harmonic entropy was obtained by summing over all non-zero vibrational modes, excluding overall translations and rotations. Calculations were performed at a temperature of 273.0 ± 3 **K**, and the first 50 eigenmodes were included in the final entropy estimation. This approach yields an approximate measure of internal entropy and serves as a complementary analysis to the relative configurational entropy method.
